# Sequence Characterization of ITS Regions of Immortelle *Helichrysum italicum* (Roth) G. Don from the East Adriatic Coast

**DOI:** 10.3390/genes14020480

**Published:** 2023-02-14

**Authors:** Matjaž Hladnik, Alenka Baruca Arbeiter, Dunja Bandelj

**Affiliations:** Faculty of Mathematics, Natural Sciences and Information Technologies, University of Primorska, Glagoljaška 8, SI-6000 Koper, Slovenia

**Keywords:** *Helichrysum*, barcodes, internal transcribed spacers, traceability, planting material

## Abstract

The immortelle (*Helichrysum italicum* (Roth) G. Don) is a typical perennial plant of natural vegetation in the Mediterranean region, and due to secondary metabolites with several biological properties (anti-inflammatory, antioxidant, antimicrobial, and anti-proliferative), it has become an important species for essential oil production, especially in the cosmetic industry. To increase the production of highly priced essential oils, it has been moved to cultivated fields. However, due to the lack of highly characterized planting material, there is a great need for genotype identification, and to provide a link with chemical profiles and geographic origin as a basis for the identification of local superior genotypes. The aims of the study were to characterize the ITS (ribosomal internal transcribed spacer) regions, ITS1 and ITS2, in samples from the East Adriatic region to determine the possibility of using these regions for plant genetic resources identification. Genetic variation was observed when comparing the ITS sequence variants of samples from the North-East Adriatic and the South-East Adriatic. Some rare and unique ITS sequence variants can be helpful for identifying specific populations from different geographical regions.

## 1. Introduction

The immortelle (*Helichrysum italicum* (Roth) G. Don (basionym = *Gnaphalium italicum* Roth)) (abbreviated as HI), known also by the synonyms ‘everlasting’ and ‘curry plant’, is a typical perennial plant of natural vegetation in the Mediterranean region, where it is traditionally used in folk medicine for healing respiratory, digestive, and skin inflammatory conditions [1,2,3,4]. Due to the strong anti-inflammatory, antioxidant, antimicrobial, and antiproliferative activities of the plant extracts and essential oil, the latter has especially become a valuable and highly prized ingredient in the perfume and cosmetic industries [4,5]. The importance of the phytochemical composition and the bioactive properties of essential oil is reflected in numerous recently performed analyses [6,7,8,9,10,11,12]. The recognized importance of the immortelle plant has led to the establishment of commercial plantations in Croatia, Slovenia, Bosnia and Herzegovina, Montenegro, and Serbia. The planting materials for these plantations usually originate from wild plants collected in natural growing sites, which are further vegetatively or generatively (via seeds) propagated in local nurseries. The overexploitation of wild populations in continental Dalmatia, and in the islands Pag and Krk (HRV) have already caused negative consequences for the natural growing sites [13]. Reduced genetic diversity, as was observed with *Tanacetum cinerariifolium* (Trevir.) Sch. Bip., another commercially interesting aromatic species, will be even more evident in the future [14].

In addition, the complex taxonomy of *Helichrysum* species, HI subspecies (four subspecies were proposed by Herrando-Moraira et al. [3]: *H. italicum* subsp. *italicum* (abbreviated as HII), *H. italicum* subsp. *microphyllum* (Willd.) Nyman (abbreviated as HIM), *H. italicum* subsp. *siculum* (Jord. and Fourr.) Galbany, L. Sáez and Benedí (abbreviated as HIS), and *H. italicum* subsp. *tyrrhenicum* (Bacch., Brullo, and Giusso) Herrando, J. M. Blanco, L. Sáez, and Galbany (abbreviated as HIT)) and the lack of taxonomic classification knowledge can lead to the propagation of incorrect plant material. The planting material of HI available in the market usually lacks the exact taxonomic classification, or is mislabeled and is sometimes offered as other species rather than HI, which is the most appreciated for essential oil production (personal observations). In such cases, the products from HI do not meet the industry requirements. The problems related to authenticity and traceability can cause economic losses in agricultural production, since the quality and biological properties of the plants are strongly influenced by genotype [15]. The latter supports the need to develop an effective traceability system that will allow for differentiation among planting material, the identification of local genotypes (for the protection of local products’ typicality and geographical origin), and the development of new cultivars with supreme quality properties, as well as the prevention of possible adulterations or the contamination of original plants with other herbs [16].

Analyses on a DNA level have become essential in identifying plant genetic resources, reference planting material, and studying genetic diversity. However, the lack of genomic data aggravates the development of reliable DNA markers. Recently, microsatellite markers were developed for HII, which showed the potential for discriminating samples from distinct geographical locations [17]. However, due to the complex taxonomic classification of *Helichrysum* species and HI subspecies, there is still a need for the development of new additional markers for species confirmation, as well as for controlling the origin and traceability of the plant material.

DNA barcodes, derived either from chloroplast or nuclear genomes, have been frequently used for species identification and phylogenetic analysis in the last decade. Among nuclear regions, internal transcribed spacers (ITS1 and ITS2) of the ribosomal RNA gene cluster (18S-5.8S-26S) were the most commonly analyzed regions across the plants [18]. The comparison of seven candidate DNA markers (*psbA-trnH*, *matK*, *rbcL*, *rpoC1*, *ycf5*, ITS1, and ITS2) from medicinal plant species revealed that the second internal transcribed spacer (ITS2) of nuclear ribosomal DNA represents the most suitable region for DNA barcoding applications. Its high discrimination ability (92.7%) was demonstrated in more than 6600 plant samples belonging to 4800 species from 753 distinct genera [19].

To increase the power of discrimination of closely related samples, ETS (external transcribed spacer) was used in combination with ITS regions in some studies with plant material belonging to the tribe Gnaphalieae (Asteraceae) [20], and species from the HAP (*Helichrysum-Anaphalis-Pseudognaphalium*) clade [21,22] and *Helichrysum* species [21,23,24,25].

It was observed that the ITS and ETS trees reflected a greater congruence with phylogenetic relationships as inferred based on morphological characters, compared to chloroplast markers [21], despite drawbacks such as intraindividual/interspecific variation and the possibility of novel recombinant types generation after species crossing (reviewed by Smissen et al. [26] and Nie et al. [20]).

Dominant markers are promising tools for studying genetic diversity, because of the simultaneous amplification of a larger number of DNA loci without prior sequence knowledge for primer development, in contrast to codominant markers. Recently, AFLP markers were used to study the genetic diversity of HI samples from 18 populations sampled along the eastern Adriatic region [14]. Based on low, though significant, genetic differentiation among the populations, extensive gene flow between populations was hypothesized. AFLP markers were also used to study differentiation between HII and HIM samples from different locations in the Mediterranean basin. The geographic structure was observed in analyzed samples, but AFLP markers did not separate the HII and HIM samples [27]. The main drawback of the dominant markers is the difficult and time-consuming scoring of genetic profiles, which limits their use for identification purposes.

The sequences of different DNA barcode regions are publicly available and could serve as a reference for the taxonomic classification and the origin identification of a defined genotype. Therefore, the aim of our study was to analyze the ITS regions of HI from the East Adriatic coast, to obtain information about the genetic variability of these ITS regions, and to determine their capability for the differentiation of samples from distinct geographical regions.

Previously mentioned studies of Galbany-Casals et al. [27] and Herrando-Moraira et al. [25], who analyzed species from the *Helichrysum pendulum* complex, and other species from *Helichrysum* sect. *Stoechadina* with ETS and *rpl32-trnL* cpDNA, also including samples of HII from Bosnia and Herzegovina, and Croatia, but these studies were not focused on the East Adriatic region.

The last aim of the study was to test the possibility of discriminating HII from the East Adriatic region from other closely related species and subspecies. To test this hypothesis, a phylogenetic analysis with additional ITS sequences from the NCBI Nucleotide database, i.e., two additional HI subspecies (HIS, HIT), *Helichrysum serotinum* subsp. *serotinum* (abbreviated as HSS) and *Helichrysum serotinum* subsp. *picardii* (Boiss. and Reut.) Galbany, L. Sáez and Benedí (abbreviated as HSP), and *Helichrysum litoreum* Guss. (abbreviated as HL) as other representatives of *H*. sect. *Stoechadina*, and selected *Helichrysum* species from *Helichrysum* sect. *Helichrysum* and *Helichrysum* sect. *Virginea*, was performed.

## 2. Materials and Methods

In total, 51 samples of HII were included in the analysis. Plants from natural habitats were sampled in Croatia and Montenegro. Two plants grown in city flower beds were sampled in Izola, Slovenia (hereby indicated as ornamental plants). In addition, four plants grown from the purchased seeds (labeled as certified seed) with a certificate that they belong to HI, were also included in the analysis (Table 1).

Morphological evaluation and distinct genetic profiles of these plants confirmed that they belong to HL (avowed substitute for *Helichrysum angustifolium* (Lam.) DC [28]) [17]. HL, compared to HI, is a very robust plant with longer leaves and many more capitula, and it is usually less glandular than HI on the abaxial sides of the leaves and on the phyllaries [1,28]. The amplified regions of plants from certified seeds of HL were compared with the obtained sequences from HII samples to identify potential polymorphisms among two different species.

Shoot tips with leaves were collected in the field and immediately preserved in a saturated NaCl-CTAB solution [29], or dried in a silica gel until DNA extraction. DNA was extracted with the protocol described by Japelaghi et al. [30], with the only difference being that phenol:chloroform:isoamyl alcohol was used instead of chloroform:isoamyl alcohol. The DNA was stored in the laboratory of the Faculty of Mathematics, Natural Sciences and Information Technologies, University of Primorska.

ITS1, 5.8S, and ITS2 regions were simultaneously amplified using the primers ITS1 and ITS4 [31]. The PCR reaction in a final volume of 50 µL contained 1× PCR buffer, 50 ng of DNA, 1.5 mM concentration of MgCl2, 0.8 mM of each dNTP, 0.5 µM of each primer, and 1.25 U of *Taq* DNA polymerase (Promega, Mannheim, Germany). The PCR protocol was as follows: a 5 min denaturation step at 94 °C, followed by 13 cycles of 35 s at 93 °C, 55 s at 53 °C, and 45 s at 72 °C. In the next 13 cycles, the denaturation and annealing steps remained the same, while the elongation step was prolonged to 59 s per cycle; in the last 9 cycles, the annealing step was 118 s. The amplification ended with the last elongation step at 72 °C for 10 min.

In order to confirm different variants of the ITS sequences in the same samples, observed as double peaks in the electropherograms, the PCR products were cloned into the pGEM^®^-T Vector (Promega, Madison, WI, USA) with the following procedure: the PCR products were excised from 1.7% agarose gel and cleaned with a Silica Bead DNA Gel Extraction Kit (Thermo Fisher Scientific, Waltham, MA, USA). pGEM^®^-T Vector ligation reactions were performed in a final volume of 5.5 μL containing 2 μL of purified PCR product, 2.5 μL of 2× Rapid Ligation Buffer, 0.5 μL of pGEM^®^-T, and 0.5 μL of ligase, and were incubated at 4 °C overnight.

The ligation mixture (3 μL) was introduced into competent *Escherichia coli* XL-10 Gold cells (Agilent Technologies, La Jolla, CA, USA) (100 μL), incubated for 20 min on ice, and transformed using the heat shock method at 42 °C for 1 min in a water bath and for 5 min on ice. A total of 900 μL of liquid SOC media (without antibiotic) was then added to the cells, followed by incubation for 45 min at 37 °C to generate the antibiotic resistance. Two different aliquots of transformation (20 μL and 70 μL) were plated onto LB agar plates containing the antibiotic, and incubated at 37 °C for one day. An LB agar medium (1 L) was made using a standard procedure, using 35 g of LB Agar High Salt2 L1706 (Duchefa, Haarlem, Neatherlands), 10 g of Daishin agar D1004 (Duchefa), 0.2 mM of IPTG (Duchefa), 40 μg ml-1 of X-GAL (Duchefa), and 150 mg l-1 of antibiotic carbenicillin (Duchefa).

For colony PCR, 10 white colonies from each LB agar plate (with the exception of sample 6) were picked with fine pipette tips, dissolved in 80 μL of TdE buffer, and heated at 96 °C for 8 min. The DNA was amplified via PCR reaction in a final volume of 20 µL containing 5 µL of DNA (dissolved in TdE buffer in the previous step), 2 μL of 10× PCR Buffer with (NH_4_)_2_SO_4_, 2 mM concentration of MgCl_2_, 0.8 mM of each dNTP, 0.5 µM of primer SP6, 0.5 µM of primer T7, and 0.5 U of *Taq* DNA polymerase (EP0401) (Thermo Fisher Scientific, Vilnius, Lithuania). After an initial denaturation of 3 min at 94 °C, the amplification protocol was 34 cycles 30 s at 94 °C, 30 s at 55 °C, and 1 min at 72 °C. The amplification was concluded with an elongation step at 72 °C for 8 min.

Amplified DNA (5 μL) was analyzed on a 1.5% agarose gel containing Midori Green (NIPPON Genetics, Düren, Germany). The rest of the reaction mixture of all successfully amplified samples was purified and used for sequencing analysis.

PCR products were cleaned with Exonuclease I (Thermo Fisher Scientific, Vilnius, Lithuania) and FastAP Thermosensitive Alkaline Phosphatase (Thermo Fisher Scientific). A DNA sequencing reaction was performed in a final volume of 7 µL, and it contained 5 µL of PCR product, 2 U of Exonuclease I, 0.5 U of FastAP Thermosensitive Alkaline Phosphatase, and 1.4 µL of 1× PCR buffer (Promega). The reaction was incubated for 45 min at 37 °C and ended at 80 °C for 15 min. The fragments were sequenced from both directions using BigDye Terminator v3.1 (Thermo Fisher Scientific) sequencing chemistry. The sequencing reaction was cleaned using the EDTA and ethanol precipitation methods, and analyzed on a 3130 Genetic Analyser (Thermo Fisher Scientific, Applied Biosystems, Tokyo, Japan).

Bases were called using KB Basecaller, implemented in Sequencing analysis software v5.1 (Applied Biosystems). The program CodonCode Aligner 6.02 (CodonCode Corporation, Dedham, MA, USA) was used to align the forward and reverse sequences. The resulting consensus sequences for each individual were aligned using ClustalW, implemented in the MEGA package 7.0 [32,33].

Dnasp v.6.12.03 [34] was used to determine the number of sequence variants and the number of variable sites (singleton variable sites and parsimony informative sites).

A barcoding gap analysis with all our sequences (HII and HL) was performed with Kimura 2-parameter (K2P) genetic distances using the barcodingR [35] package.

A network with ITS1-5.8S-ITS2 sequence variants was created with the TCS method [36], implemented in the PopArt software [37]. An input Nexus file was prepared with MEGA package 7.0. HII sequences (accession numbers from KJ159118.1 to KJ159126.1) were included in the construction of the network as well. For the network analysis, sampling locations were grouped into the North-East Adriatic and the South-East Adriatic. Sequence variants of plants from certified seeds and from ornamental plants were considered separately. The distribution of sequence variants across geographical locations was presented with a map, generated with R version 4.2.1, and R packages maps (3.4.0) and mapplots (1.5.1). Newly identified sequence variants of HII have been deposited in the NCBI Nucleotide database (accession numbers from OP874600 to OP874610), whereas sequence variants identified only at HL were assigned to GenBank accession numbers from OQ330863 to OQ330867.

Phylogenetic analysis was performed with ITS sequence variants (concatenated ITS1 and ITS2 regions; 5.8S rDNA sequence was excluded from the analysis) of HII obtained in this study, and with additional sequences of selected species/subspecies belonging to *H.* sect. *Stoechadina* (*Helichrysum crassifolium* D. Don (abbreviated as HC), *Helichrysum heldreichi* Boiss. (abbreviated as HH), HIS, HIT, *Helichrysum massanellanum* Herrando, J. M. Blanco, L. Sáez and Galbany (abbreviated as HM), HSS and HSP, *Helichrysum stoechas* (L.) Moench (abbreviated as HSt)), *H*. sect. *Helichrysum* (*Helichrysum thianschanicum* Regel (abbreviated as HT) and *Helichrysum orientale* (L.) Vaill. (abbreviated as HO)), and *H.* sect. *Virginea* (*Helichrysum sibthorpii* Rouy (abbreviated as HSi)), which were downloaded from the NCBI Nucleotide database. Species from *H.* sect. *Helichrysum* and *H*. sect. *Virginea* were included as a control for our study, since they formed different phylogenetic clades in previous studies [21,24]. Among the existing HI subspecies with ITS sequences available in the NCBI Nucleotide database, HII, HIT, and HIS were included in the analysis. Most of the sequences belonged to HII from Corsica (FR). However, in view of the revised classification of the HI subspecies [3] and after additional verification of the origin of the sequenced samples deposited in the NCBI database, some taxonomic annotations were updated, considering the latest taxonomic classification. Sample HM244710.1 originated from Corsica (source: GenBank); thus, its previous classification, HIM, was corrected to HIT, since the subsp. *microphyllum* is restricted to Crete (GR) only [3]. In addition, the sequence AY445196.1 originally annotated to HI and collected in Sicily (IT) [23] represents HIS. The new classification assumes that HIS is endemic to Sicily [3]. Another sample, AY445195.1, collected on Massanella massif (Majorcan mountain) [23], previously classified as HIM, was assigned to a newly described species, HM [3].

The best available model of DNA substitution for the data was identified with the Akaike Information Criterion (AIC), as implemented in MrModeltest2 v2.4 [38]. A HKY model with the gamma distribution (G) shape parameter 0.1770 was identified as the most appropriate model. Phylogenetic analysis using the Bayesian inference (BI) and Maximum Likelihood (ML) was performed with MrBayes v3.2.7a [39] and PhyML v3.3 [40], respectively. Four Monte Carlo Markov chains were run simultaneously for 5 × 10^6^ generations, with the resulting trees sampled every 500 generations. Bayesian posterior probabilities (BPP) were used to assess the branch support of the BI tree. Trace files were imported in TRACER v1.7.1 to examine the trace plots and effective sample size (ESS) estimates [41]. The following flags were used for the phyml analysis: -d ‘nt’ -b 1000 -m HKY85 -t 1.7025 -a 0.1770 --leave_duplicates. Branch support in the ML tree was estimated using 1000 bootstrap replicates. The resulting trees were compared using a Shimodaira–Hasegawa (SH) test [42] implemented in PAUP v4.0a168 [43], based on likelihood scores. The following command was used: Lscore all/nst = 6 rmatrix = estimate basefreq = estimate rates = gamma shape = estimate pinvar = estimate Shtest = yes bootreps = 1000. Trees were rooted with species *Helichrysum litorale* Bolus (KT865507).

## 3. Results and Discussion

Immortelle is a relatively new plant in agricultural cultivation, enabling the production of highly prized essential oil and the development of products with high added value in the cosmetic industry. According to our own experience, there is confusion regarding the correct naming and labeling of seedlings in the market. The main reasons for this are the abundance of different *Helichrysum* species and HI subspecies present in the Mediterranean region, the complex taxonomical classification, the presence of hybrids, and the transfer of planting material between countries.

In this study, 51 HII samples, including 49 samples from their natural growing sites in the North-East Adriatic and the South-East Adriatic regions, and two ornamental samples from city flower beds, were used for the amplification of the ITS1-5.8S-ITS2 regions to determine whether the geographical origins of the samples can be identified based on polymorphisms of the ITS sequences. The geographical region may have an influence on the development of unique populations with special genetic backgrounds, which can be reflected in the quality of immortelle products. From this point of view, it is important to obtain information about genetic resources for the successful introduction of plants in the agriculture ecosystem. In addition, four samples obtained from purchased seeds with a certificate that they belong to HI, but later determined as HL [17], were also included to test the ability of ITS loci to detect not true-to-type plant material. HL is present in the East Adriatic region, and it grows together with HI in certain areas, leading to the development of intermediate specimens [1].

An examination of the electropherograms revealed the presence of up to two double peaks at 27 samples. After the reamplification of PCR products with a DNA cloning technique, up to four different sequence variants were identified in one sample. In total, 93 sequences (considering all sequence variants per sample) were obtained in the analyzed samples.

An intra-individual variation of ITS sequences was commonly observed [44]. In addition, intraindividual variation was observed for the ETS marker [25], which is a part of the same transcriptional unit.

**Figure 1:**
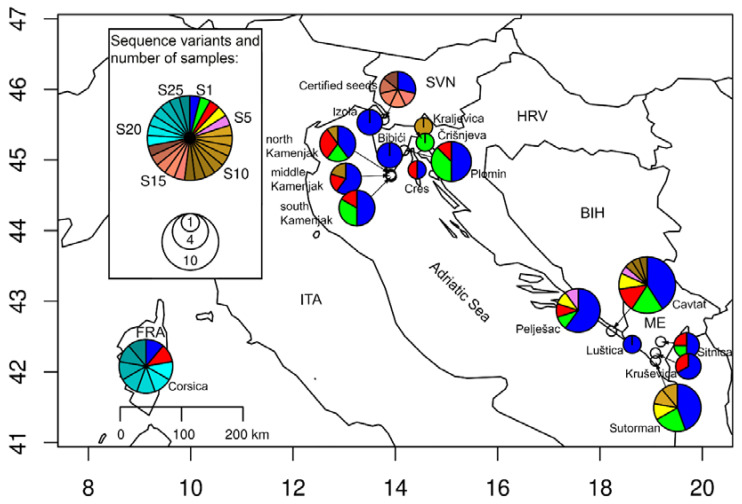
Distribution of identified ITS1-5.8S-ITS2 sequence variants across the North-East and South-East Adriatic regions. Sequences from Corsica were obtained from the NCBI Nucleotide database. The radii of pie charts indicate the number of samples per location (from 1 to 10), whereas the sizes of the slices present the relative abundances of sequence variants. The most frequently observed sequence variants, labeled 1 to 5, were marked with distinct colors, whereas sequences identified at single samples were marked with similar color shades, based on their origins (S6 to S13 North-East and South-East Adriatic region, S14 to S18 certified seeds (identified as HL) and S19 to S25 GenBank sequences from Corsica plants).

After the multiple sequence alignment, 25 bp were removed from the beginning of the ITS1 region, since the first few bases were not called with adequate quality scores in a few samples. However, the entire ITS2 region was sequenced in all samples. In total, 18 ITS sequence variants with 12 polymorphic sites were identified (Supplementary Table S1). In ITS1, three singleton variable sites and five parsimony informative sites were observed, whereas in the ITS2 region, there were two singleton variable sites and one parsimony informative site. One sample from certified seed showed a mutation in the 5.8S gene, and an insertion of 2 bp was observed in one sample from Kamenjak in the ITS2 region. The distribution of the ITS1-5.8S-ITS2 sequence variants across sampling geographical regions is shown in Figure 1.

**Figure 2:**
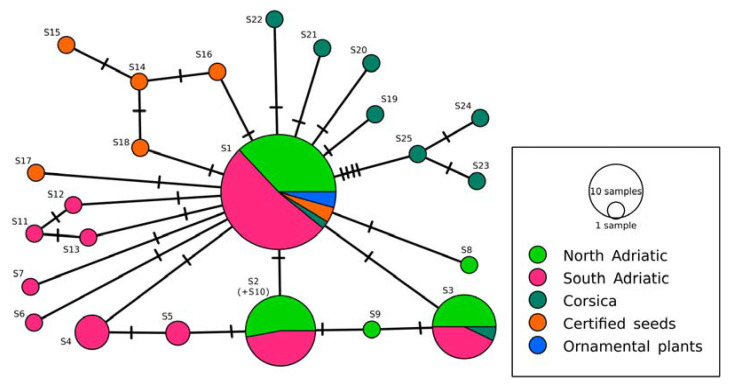
HII TCS network with ITS1-5.8S-ITS2 sequence variants. Sequences with labels from S1 to S5 indicate the most frequently observed sequence variants (S1, S2, S3, S4, and S5 were identified in 46, 17, 14, 4, and 2 samples, respectively). Hatch marks present the number of mutations. Sequences from Corsica were downloaded from the NCBI Nucleotide database (accession numbers from KJ159118 to KJ159126).

A TCS network analysis was performed to identify relationships among the ITS1-5.8S-ITS2 sequence variants (Figure 2). Sequence variant S10 with the insertion was considered as S2, since PopArt masked gaps.

The most frequently represented sequence variant S1 was observed in all geographical regions, as well as in plants from certified seeds (previously classified as HL) and in ornamental plants. Fifteen sequences were derived from S1 (including sequences from Corsica’s samples) with one mutational step. Sequence variant S2 was observed in samples from the North-East and South-East Adriatic regions only. Sequence S3 was observed in samples from the East Adriatic region and Corsica, whereas sequences S4 and S5 were identified only in the South-East Adriatic region. Twelve sequence variants were observed only once in different samples (seven sequence variants were identified only once in the samples from the South-East Adriatic, and two sequence variants were observed only once in the samples from the North-East Adriatic, respectively). Five unique sequence variants (S14–S18) were observed in samples from certified seeds (HL), whereas sequences S19–S25 were identified in Corsica only.

**Figure 3:**
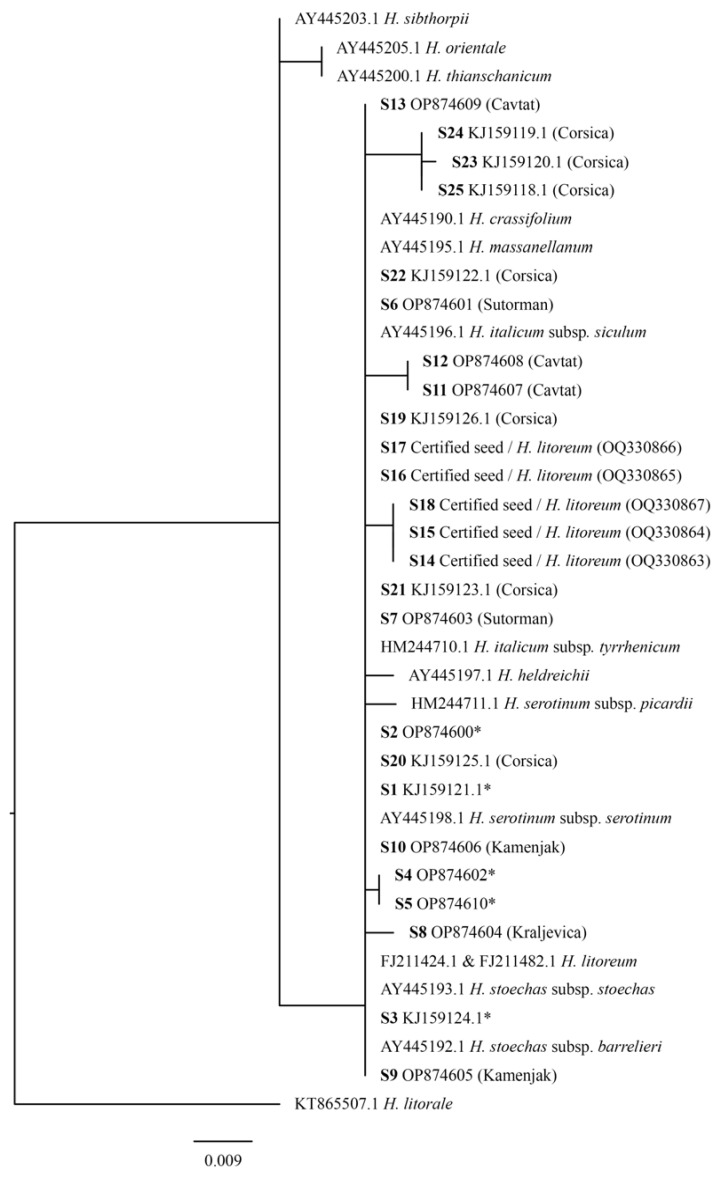
Maximum likelihood tree with the ITS sequences. *H. litorale* species was used as a root. Sequence variants S1 to S5 of HII (marked with an asterix) were found in two or more samples from different locations (sequences with GenBank accession numbers represent a reference sequence), whereas the other ITS sequence variants were found in separate samples.

Bayesian phylogenetic (BP) and maximum likelihood (ML) analyses were performed in order to infer the relationship of HII ITS sequence variants from distinct geographical locations, and the relationship to other selected closely related species from the *Helichrysum* genus. The SH test did not reveal a significant difference between the BP and ML tree (the ML tree is presented in Figure 3).

Based on ML phylogenetic analysis, HT and HO (belonging to *H*. sect. *Helichrysum*) were separated from HSi (belonging to *H*. sect. *Virginea*), whereas all HI subspecies, as well as *H. serotinum* subspecies, *H. stoechas* subspecies, HH, HL, HM, and HC were placed in the same clade, with subclades and leaves being presented as multifurcations. All previously mentioned taxa (except HT, HO, and HSi) belong to a monophyletic *H*. sect. *Stoechadina*, which generally has a western-central Mediterranean distribution [21,24].

The discrimination of species from the *H.* sect. *Stoechadina* was not observed since HII ITS sequences were in groups with different *Helichrysum* species, and the same ITS sequences were identified in different taxonomic units (HC (AY445190.1), HIS (AY445196.1), HSt subsp. *stoechas* (AY445193.1), HM (AY445195.1), HL (FJ11424.1 and FJ211482.1), and sequence S1 (including HL from certified seeds (Table S1)), which was found in samples from all locations). Sequences of HSt subsp. *barrelieri* (AY445192.1) and HSS (AY445198.1) were duplicates of the sequence S2, characteristic for the North and South Adriatic.

A low resolution of the nrDNA phylogenies, based on ITS or ETS alone, was reported for the Mediterranean-Asiatic *Helichrysum* group [24]. In addition, morphologically distinct taxa from close geographic origins were grouped together. This could be a consequence of historical and contemporary hybridizations [1,21,25]. Galbany-Casals et al. [27] indicated that specimens from some localities appear to be intermediate between HII and either HIM or HIS. The same authors performed a comparison of the chloroplast *psbA-trnH* sequence barcode of 81 samples of different subspecies of HI (subsp. *italicum*, subsp. *microphyllum*, and subsp. *siculum*) and other members of sect. *Stoechadina* (species HL, HSP, and HSS), and showed that some chloroplast haplotypes were shared across species (e.g., haplotype G was identified at HII, HIM, HL, and HSS). The same was observed in the group of the New Zealand endemic species of Gnaphalieae, where the chloroplast *psbA-trnH* marker was used, and the obtained haplotypes did not correspond with the taxonomic units [26].

In addition, AFLP markers and ITS sequences, together with the morphological data, supported the existence of hybrids between HO and HSt [45].

However, since the region-specific ITS sequence variants were observed in our study, the results were in agreement with Galbany-Casals et al. [24], as they also observed a correlation of nrDNA markers with geographic distribution. Additionally, Herrando-Moraira et al. [25] observed that in the case of ETS, most of the variation (83.9%) could be attributed to the differences between populations, which supported the presence of the phylogeographical structure for this marker. Region-specific ITS sequence variants could be supported with the recently published study of Ninčević et al. [14], which showed a clustering of HI populations with AFLP markers into two distinct clusters, i.e., (1) populations from the north (Kvarner Bay, HRV) and (2) populations from the central and southern parts of Dalmatia (HRV).

With the ITS sequence polymorphisms, we were not able to discriminate the HI subspecies. The latter could be explained with the hypothesis of Galbany-Casals et al. [27], where due to the widespread distribution of HI, it is probably not possible to define discrete populations because of gradual clinal variation.

Despite the low discrimination resolution observed among the analyzed sequences, distinct ITS1-5.8S-ITS2 sequence variants were observed in two plants obtained from certified seeds (HL). However, two plants shared a sequence variant that was identical to the most common sequence variant (S1), and the same was noticed for the ITS reference sequences of HL from the NCBI Nucleotide database. A barcoding gap analysis with our sequences (HII and HL) with Kimura 2-parameter (K2P) genetic distances revealed no gap between intraspecies and interspecies genetic distances. One of the reasons for this could be the different number of sequence variants compared (HII: 86 vs. HL: 7) in this study. Therefore, an additional analysis of the plant material is needed to confirm that the obtained distinct sequence variants are characteristic of HL. It can be mentioned that HL and HI can be better differentiated using microsatellites, as a comparison of their DNA profiles revealed 8 unique alleles and 14 unique allelic combinations at multiple loci in the HL sample [17].

## 4. Conclusions

The study demonstrated that the same ITS sequences are shared by different HI individuals from different geographic regions, and even by different species of *H.* sect. *Stoechadina*, making this marker not useful as a barcode for the differentiation of *Helichrysum* species. However, some rare, localized, and uncommon sequence variants can be helpful to identify specific populations or geographic variants. In addition, new ITS1-5.8S-ITS2 regions of 11 HII and 5 sequence variants of HL from the East Adriatic region have been deposited in the NCBI Nucleotide database. In the future, it is expected that an increased number of accessible sequences in the NCBI Nucleotide database and the new markers developed for HI will contribute to a better resolution of the species and subspecies of this important aromatic plant.

## Figures and Tables

**Table 1:** List of sampling locations and number of HII samples from each collection, including samples from certified seeds (HL).

Sampling Location	Country	Origin	Number of Samples
Bibići	Croatia	Natural habitat	2
Cavtat	Croatia	Natural habitat	10
Cres island	Croatia	Natural habitat	1
Črišnjeva	Croatia	Natural habitat	1
Kamenjak peninsula (north, middle, and south parts)	Croatia	Natural habitat	11
Kraljevica	Croatia	Natural habitat	1
Plomin	Croatia	Natural habitat	5
Pelješac peninsula	Croatia	Natural habitat	6
Border crossing point Sitnica	Montenegro	Natural habitat	2
Kruševica	Montenegro	Natural habitat	2
Luštica peninsula	Montenegro	Natural habitat	1
Sutorman	Montenegro	Natural habitat	7
Izola	Slovenia	Ornamental	2
Certified seeds	Slovenia	Certified seeds	4

## Data Availability

The representative sequence variants have been deposited in the NCBI Nucleotide database under accession numbers OP874600 to OP874610 (*H. italicum* subsp. *italicum*), and from OQ330863 to OQ330867 (*H. litoreum*).

## Supplementary Materials

The following supporting information can be downloaded at: https://www.mdpi.com/article/10.3390/genes14020480/s1, Table S1. List of identified mutations in the observed sequence variants.
