# Supplementary material for: Sequence Characterization of ITS Regions of Immortelle Helichrysum italicum (Roth) G. Don from the East Adriatic Coast
Source: Genes (Basel). 2023 Feb 14;14(2):480. doi: 10.3390/genes14020480 (PMC9957125; doi:10.3390/genes14020480)
Supplement: Supplementary file 1 [file genes-14-00480-s001.zip › genes-2161346-supplementary.pdf]

Table S1. List of identified mutations in the observed sequence variants.

	ITS1												5.8S rRNA	ITS2										
	$48^{s,*}$	51	54	60^{p}	88	89^{s}	159	188^{p}	193^{p}	194^{s}	217^{p}	234^{p}	389	425	433^{p}	443^{s}	470	478-479	486	520	560^{s}	598	624
S1[a]	G	A	T	A	C	T	C	G	G	G	G	C	T	G	C	A	T	-	C	C	C	G	C
S2	.	.	.	.	.	.	.	A	.	.	.	.	.	.	.	.	.	-	.	.	.	.	.
S3	.	.	.	G	.	.	.	.	.	.	.	.	.	.	.	.	.	-	.	.	.	.	.
S4	.	.	.	.	.	.	.	.	.	.	.	.	.	.	T	.	.	-	.	.	.	.	.
S5	.	.	.	.	.	.	.	A	.	.	.	.	.	.	T	.	.	-	.	.	.	.	.
S6	.	.	.	.	.	.	.	.	.	.	.	.	.	.	.	.	.	-	.	.	A	.	.
S7	.	.	.	.	.	C	.	.	.	.	.	.	.	.	.	.	.	-	.	.	.	.	.
S8	T	.	.	.	.	.	.	.	.	.	.	.	.	.	.	.	.	-	.	.	.	.	.
S9	.	.	.	G	.	.	.	A	.	.	.	.	.	.	.	.	.	-	.	.	.	.	.
S10	.	.	.	.	.	.	.	A	.	.	.	.	.	.	.	.	.	TC	.	.	.	.	.
S11	.	.	.	.	.	.	.	.	.	.	A	G	.	.	.	.	.	-	.	.	.	.	.
S12	.	.	.	.	.	.	.	.	.	.	.	G	.	.	.	.	.	-	.	.	.	.	.
S13	.	.	.	.	.	.	.	.	.	.	A	.	.	.	.	.	.	-	.	.	.	.	.
S14	.	.	.	.	.	.	.	.	A	.	.	.	C	.	.	.	.	-	.	.	.	.	.
S15	.	.	.	.	.	.	.	.	A	C	.	.	C	.	.	.	.	-	.	.	.	.	.
S16	.	.	.	.	.	.	.	.	.	.	.	.	C	.	.	.	.	-	.	.	.	.	.
S17	.	.	.	.	.	.	.	.	.	.	.	.	.	.	.	T	.	-	.	.	.	.	.
S18	.	.	.	.	.	.	.	.	A	.	.	.	.	.	.	.	.	-	.	.	.	.	.
S19	.	.	.	.	.	.	.	.	.	.	.	.	.	.	.	.	.	-	.	.	.	.	T
S20	.	.	.	.	.	.	.	.	.	.	.	.	.	.	.	.	.	-	.	T	.	.	.
S21	.	.	.	.	.	.	.	.	.	.	.	.	.	.	.	.	C	-	.	.	.	.	.
S22	.	.	.	.	.	.	.	.	.	.	.	.	.	.	.	.	.	-	T	.	.	.	.
S23	.	G	C	.	.	.	T	.	.	.	.	.	.	A	.	.	.	-	.	.	.	A	.
S24	.	G	C	.	T	.	T	.	.	.	.	.	.	.	.	.	.	-	.	.	.	A	.
S25	.	G	C	.	.	.	T	.	.	.	.	.	.	.	.	.	.	-	.	.	.	A	.

* The numbers in the columns indicate the positions of the mutations based on the full length ITS1 sequence (identified after alignment to the ITS1 sequence (FJ211422.1)). Positions after insertion are elevated according to the size of the insertion. The completeness of the ITS2 region was verified with the alignment to the FJ211480.1. In this study, sequence variants from S1 to S18 were observed whereas ITS sequences downloaded from the NCBI Nucleotide database were included with labels from S19 to S25. Singletons and parsimony informative sites in ITS regions are indicated with a superscript "s" and "p", respectively. Sites without superscript labelling were polymorphic only in sequences from the NCBI Nucleotide database.

[a]List of samples with observed haplotypes: **S1** (46 samples): KJ159121.1, 02_Sitnica_1, 03_Sitnica, 04__Kruševica_1, 05_Kruševica, 06_Sutorman, 07_Sutorman, 11_Sutorman, 12_Sutorman_1, 18_Plomin, 19_Plomin_2, 21_Plomin_2, 23_Plomin_2, 31_Kamenjak_1, 32_Kamenjak_2, 33_Kamenjak_1, 35_Kamenjak_2, 36_Kamenjak, 39_Kamenjak_1, 40_Kamenjak_1, 42_Kamenjak_2, 43_Kamenjak_1, 44_Kamenjak, 47_Bibiči, 48_Cavtat_2, 49_Cavtat_2, 51_Cavtat_4, 52_Cavtat_2, 55_Cavtat_1, 56_Cavtat_3, 57_Cavtat, 58_Cavtat, 60_Cavtat_1, 63_Pelješac, 64_Pelješac, 66_Pelješac, 67_Pelješac_1, 71_Pelješac, 72_Pelješac_1, 77_Certified_seed, 79_Certified_seed, 83_Izola, 84_Izola, 85_Bibici, 87_Luštica, 91_Cres_1; **S2** (16 samples): 02_Sitnica_2 (OP874600), 13_Sutorman, 14_Sutorman_2, 19_Plomin_1, 21_Plomin_1, 23_Plomin_1, 32_Kamenjak_1, 35_Kamenjak_1, 41_Kamenjak, 42_Kamenjak_1,

48_Cavtat_1, 51_Cavtat_1, 52_Cavtat_1, 56_Cavtat_1, 67_Pelješac_2, 95_Črišnjeva; **S3** (14) KJ159124.1, 02_Sitnica_4, 04_Kruševica_2, 25_Plomin, 31_Kamenjak_2, 32_Kamenjak_4, 33_Kamenjak_2, 39_Kamenjak_2, 43_Kamenjak_2, 50_Cavtat, 55_Cavtat_2, 60_Cavtat_2, 72_Pelješac_2, 91_Cres_2; **S4** (4): 12_Sutorman_2 (OP874602), 51_Cavtat_2, 56_Cavtat_2, 67_Pelješac_3; **S5** (2): 51_Cavtat_3 (OP874610), 67_Pelješac_4; **S6**: 10_Sutorman (OP874601); **S7**: 14_Sutorman_1 (OP874603); **S8**: 16_Kraljevica (OP874604); **S9**: 32_Kamenjak_3 (OP874605); **S10**: 40_Kamenjak_2 (OP874606); **S11**: 49_Cavtat_1 (OP874607); **S12**: 49_Cavtat_3 (OP874608); **S13**: 49_Cavtat_4 (OP874609); **S14**: 76_Certified_seed_1 (OQ330863); **S15**: 76_Certified_seed_2 (OQ330864); **S16**: 76_Certified_seed_3 (OQ330865); **S17**: 80_Certified_seed_1 (OQ330866); **S18**: 80_Certified_seed_2 (OQ330867); **S19**: KJ159126.1; **S20**: KJ159125.1; **S21**: KJ159123.1; **S22**: KJ159122.1; **S23**: KJ159120.1; **S24**: KJ159119.1; **S25**: KJ159118.1 (Labels indicate sample number, geographical location, and sequence variant identifier of the same plant if more than one sequence variant was observed).
